# A Nutrition-Related Factor-Based Risk Stratification for Exploring the Clinical Benefits in the Treatment of Patients With Locally Advanced Esophageal Squamous Cell Carcinoma Receiving Definitive Chemoradiotherapy: A Retrospective Cohort Study

**DOI:** 10.3389/fnut.2022.896847

**Published:** 2022-08-04

**Authors:** Yilin Yu, Haishan Wu, Jianjian Qiu, Dongmei Ke, Yahua Wu, Mingqiang Lin, Tianxiu Liu, Qunhao Zheng, Hongying Zheng, Jun Yang, Zhiping Wang, Hui Li, Lingyun Liu, Qiwei Yao, Jiancheng Li, Wenfang Cheng, Xiaohui Chen

**Affiliations:** ^1^Department of Thoracic Surgery, Fujian Medical University Cancer Hospital, Fujian Cancer Hospital, Fuzhou, China; ^2^College of Clinical Medicine for Oncology, Fujian Medical University, Fuzhou, China; ^3^The Graduate School, Fujian Medical University, Fuzhou, China; ^4^Department of Radiation Oncology, Fujian Medical University Cancer Hospital, Fujian Cancer Hospital, Fuzhou, China

**Keywords:** esophageal squamous cell carcinoma, body mass index, prognostic nutritional index, nutritional indices, risk stratification, definitive chemoradiotherapy

## Abstract

**Objective:**

No study has reported the risk stratification of BMI and PNI in patients with locally advanced esophageal squamous cell carcinoma (ESCC) undergoing definitive chemoradiotherapy (dCRT). This study aimed to construct a risk stratification to guide the treatment of ESCC following dCRT.

**Methods:**

A total of 1,068 patients with locally advanced ESCC who received dCRT were retrospectively analyzed. The impacts of clinicopathological factors on overall survival (OS) and progression-free survival (PFS) were analyzed. Besides, the novel prognostic indices of pre-therapeutic nutritional index (PTNI) and prognostic index (PI) were developed.

**Results:**

The median follow-up period of OS and PFS were 22.9 and 17.4 months, respectively. The high body mass index (BMI) group had better 5-year OS and PFS (36.4 and 34.0%) than the low BMI group (18.8 and 17.2%). The high prognostic nutritional index (PNI) group also had better 5-year OS and PFS (33.4 and 30.9%) than the low PNI group (17.5 and 17.2%). Multivariate Cox regression analysis showed that BMI and PNI were independent prognostic factors for OS and PFS. Based on nutritional indices, patients were categorized into the low-risk (PTNI = 1), medium-risk (PTNI = 2), and high-risk (PTNI = 3) groups with 5-year OS rates of 38.5, 18.9, 17.5%, respectively (*p* < 0.001) and 5-year PFS rates of 35.8, 17.6, 16.8%, respectively (*p* < 0.001). Besides, we also constructed a prognostic index (PI) for OS and PFS which was calculated based on statistically significant factors for predicting OS and PFS. The results revealed that the high-risk group had worse OS and PFS than the low-risk group (*p* < 0.001). Finally, RCS analysis demonstrated a non-linear relationship between the PNI, BMI, and survival for patients with ESCC. The death hazard of PNI and BMI sharply decreased to 41.8 and 19.7.

**Conclusion:**

The decreased pre-therapeutic BMI and PNI levels were associated with a worse survival outcome. BMI and PNI are readily available and can be used to stratify risk factors for locally advanced ESCC patients undergoing dCRT. The novel risk stratification may help to evaluate patients’ pre-therapeutic status and guide dCRT for locally advanced ESCC patients.

## Introduction

Esophageal carcinoma (EC) is one of the most prevalent and aggressive malignant neoplasms, with a poor prognosis (1). It is ranked as the world’s sixth-leading cause of cancer-related mortality, accounting for more than 500,000 deaths every year (2). Despite the advancements in comprehensive treatments such as surgery, radiotherapy, and chemotherapy that have been achieved, the prognosis for EC remains poor. Survival outcomes are still undesirable even though definitive chemoradiotherapy (dCRT) remains the primary treatment option for patients with inoperable EC. Consequently, a pre-therapeutic risk assessment of patients is needed to predict outcomes after treatment.

Body mass index (BMI) is a risk factor for EC (3, 4). Previous studies have indicated that BMI can potentially represent comprehensive information on body composition status, especially in cancer patients (5). The decreased BMI is an available marker for sarcopenia in patients with ESCC (6). Besides, the decreased BMI is associated with lower immunity (7). A study found that a low BMI is associated with an increased risk and also increases mortality in esophageal squamous cell carcinoma (ESCC) patients (4). Therefore, survival advantage in patients with high BMI may be related to their greater nutritional reserves. Nevertheless, emerging evidence suggests that BMI alone is insufficient to accurately evaluate the prognosis in patients with ESCC. These findings demonstrated BMI’s limited value. It is worth noting that ESCC patients usually have a unique body composition, which is significantly different from that of patients with other malignant tumors (8). Another risk factor for EC is the prognostic inflammatory and nutritional index (PNI), which can be easily determined using peripheral lymphocyte count and serum albumin level (9, 10). Several studies have also linked BMI and PNI to the survival outcome in patients with gastric cancer (11), oral cancer (12), and non-small cell lung cancer (13). These findings support the use of BMI and PNI in predicting the survival outcome in patients with ESCC, although their impact on patients with ESCC is still unclear.

Despite previous studies showing an association between BMI, PNI, and prognosis in patients with EC (14–20), the impact of pre-therapeutic BMI and PNI on survival outcomes in patients with ESCC who received dCRT remains scarce and contentious. Also, most studies are based on patients undergoing surgery. There is no known study on the risk stratification of BMI and PNI in patients with ESCC who received dCRT. Reasonable risk stratification of nutritional indices is therefore needed to help in treating patients with ESCC following dCRT. Unfortunately, there is still no reliable predictive system for ESCC patients after dCRT. Considering the current status, this study aims to perform risk stratification of nutritional indices to investigate the predictive index and classify patients at different risks that may benefit from dCRT.

## Materials and Methods

### Patient Population

A retrospective study was carried out at the Fujian Provincial Cancer Hospital. Patients with locally advanced ESCC who underwent dCRT at the radiotherapy department were included between January 2010 and December 2020. The inclusion criteria were as follows: (A) Histopathologic confirmation of ESCC; (B) aged ≥18 years; (C) Karnofsky Performance Status ≥70 points; (D) had not undergone surgery; and (E) no distinct metastasis or multiple primary diagnoses. The exclusion criteria included: (A) Patients with severe renal and hepatic disorders; (B) no available data were present; and (C) patients who received two–dimensional conventional radiotherapy (2D-CRT). All the patients were diagnosed with locally advanced ESCC (stage II–IVA). Before the first treatment, the patients’ height and weight were measured. The blood biochemical data were collected 1 week before treatment. The eighth edition of the tumor, node, and metastasis (TNM) classification was used for clinical staging in all patients. The study was performed per the Declaration of Helsinki guidelines and was approved by the Ethics Committee of Fujian Provincial Cancer Hospital.

### Radiotherapy and Chemotherapy

Six megavolt beams were used in the radiotherapy regimen. Computed tomography (CT)-based radiation planning, three-dimensional-conventional radiotherapy (3D-CRT), and intensity-modulated radiotherapy (IMRT) were used in the patients. In total, 95% of the planning target volume (PTV) dose was 50–70 Gy (1.8–2 Gy per fraction, 25–35 fraction). All of the 1,068 eligible patients had received 0–9 courses of sequential or concurrent chemotherapy. The regimens of chemotherapy were based on platinum, including (A) paclitaxel d1 or docetaxel d1 + cisplatin d2 or lobaplatin d2 or nedaplatin d2 or carboplatin AUC 2 d2; (B) cisplatin d2 + 5–fluorouracil (5-FU) d1–2.

### Determination of the Nutritional Index and Inflammatory Index

BMI (kg/m^2^) was calculated by dividing weight (in kilograms) by the square of height (in meters). The PNI was calculated by the serum albumin (ALB) level (g/L) + 5 multiplied by the absolute lymphocyte count. The platelet-to-lymphocyte ratio (PLR) was calculated by dividing the absolute platelet count by the absolute lymphocyte count. The absolute neutrophil count was divided by the absolute lymphocyte count to obtain the neutrophil-to-lymphocyte ratio (NLR). The lymphocyte-to-monocyte ratio (LMR) was obtained by dividing the absolute lymphocyte count by the absolute monocyte count. The absolute platelet count was multiplied by NLR to obtain the systemic immune-inflammation index (SII).

### Generating Prognostic Index for Overall Survival and Progression-Free Survival

The PI predicting a risk of survival was calculated by the Cox regression model. The PI was obtained by summating each significant factor weighted by the hazard ratio. The PI for predicting OS and PFS was generated by multiplying weighting factor (b coefficient) to each variable. Based on the result of calculated risk score, the patients were divided into high-risk and low-risk groups.

### Follow-Up and Endpoint Definition

All patients were followed up to obtain data on disease progression and survival status every 3 months during the first year, every 6 months in the following 2 years, and once a year after that. Routine review items involved physical examination, biochemistry, blood tests, tumor markers, barium esophagography, gastrointestinal endoscopy, CT, or positron emission tomography-CT (PET-CT). Information about disease progression and survival status was updated until April 2021. The endpoints of the study were PFS and OS. The PFS is defined as the time from pathological diagnosis to tumor progression, death, or the last follow-up. The OS is defined as the time from pathological diagnosis to death or the last follow-up. The follow-up information came from telephone interviews and/or patients’ clinical charts.

### Statistical Analysis

Statistical analysis was done using R software (ver. 4.0.2) and SPSS software (ver. 26.0) for Windows. The X-tile application^1^ was used to establish the optimal cutoff values for radiotherapy (RT) dose, BMI, PNI, PLR, NLR, LMR, and SII. When comparing categorical data, Chi-square or Fisher’s exact test was employed. To compare continuous variables, the Mann-Whitney *U*-test was used. The survival curves were constructed using the Kaplan-Meier method. The Cox regression model was carried out for univariate and multivariate analysis. All variables with *p* < 0.05 in univariate were included in multivariate analysis to identify independent prognostic factors. The rms R package was used to construct a nomogram. The Hmisc R package was used to obtain the C-index and calibration curves. A bootstrap approach with 1,000 resamples was used to calculate the C-index, which was then used to determine the discrimination ability of the nomogram. In addition, Restricted Cubic Splines (RCS) was used to examine the relationship between factors and survival outcomes by the rms R package. The correlation between nutritional indices was analyzed using Spearman’s correlation analysis. A two-tailed *p* < 0.05 was considered statistically significant.

## Results

### Patient Characteristics According to Body Mass Index and Prognostic Nutritional Index

The clinicopathological characteristics of the 1,068 patients are shown in Table 1. All enrolled patients had ESCC. There were 769 males (72.0%) and 299 female patients (28.0%) involved in the study. The clinical stage was II for 225 patients (21.1%), III for 324 patients (30.3%), and IV for 519 patients (48.6%). The median BMI and PNI values were 21.4 (range, 13.3–34.6) and 47.9 (range, 30.3–76.3), respectively. There were 313 (29.3%) and 755 (70.7%) patients with low (≤19.7) and high (>19.7) BMI, respectively. There were 150 (14.0%) and 918 (86.0%) patients with low (≤41.8) and high (>41.8) PNI, respectively. Most patients received chemotherapy (71.8%). Only a small percentage of patients had an RT dose ≤59.5 Gy (17.1%). The optimal cutoff values for RT dose, BMI, PNI, PLR, NLR, LMR, and SII were calculated to be 59.5Gy, 19.7, 41.8, 195.29, 4.56, 3.73, and 918, respectively.

**TABLE 1:** Patient characteristics of 1068 patients with locally advanced ESCC according to BMI and PNI.

Clinicopathologic variable		N	BMI ≤19.7	BMI >19.7	p	N	PNI ≤41.8	PNI >41.8	p
N		1,068	313	755		1,068	150	918	
Age (years)					0.345				0.172
	≤ 70	690	195	495		690	89	601	
	>70	378	118	260		378	61	317	
Gender					0.049				0.625
	Male	769	239	530		769	111	658	
	Female	299	74	225		299	39	260	
Tumor location					0.073				0.246
	Cervical	106	28	78		106	11	95	
	Upper thoracic	298	72	226		298	43	255	
	Middle thoracic	551	179	372		551	74	477	
	Lower thoracic	113	34	79		113	22	91	
Weight loss					0.062				0.448
	Yes	514	165	349		514	77	437	
	No	554	148	406		554	73	481	
Chemotherapy					0.006				0.028
	Yes	767	206	561		767	96	671	
	No	301	107	194		301	54	247	
RT dose (Gy)					0.490				0.068
	≤59.5	183	58	125		183	34	149	
	>59.5	885	255	630		885	116	769	
T stage					0.029				0.961
	T2	75	22	53		75	10	65	
	T3	519	133	386		519	72	447	
	T4	474	158	316		474	68	406	
N stage					0.208				0.130
	N0	299	77	222		299	33	266	
	N1	465	134	331		465	69	396	
	N2	234	79	155		234	33	201	
	N3	70	23	47		70	15	55	
TNM stage					0.002				0.334
	Stage II	225	45	180		225	25	200	
	Stage III	324	99	225		324	50	274	
	Stage IVA	519	169	350		519	75	444	
PLR					<0.001				<0.001
	≤195.29	866	225	641		866	68	798	
	>195.29	202	88	114		202	82	120	
NLR					<0.001				<0.001
	≤4.56	953	256	697		953	98	855	
	>4.56	115	57	58		115	52	63	
LMR					<0.001				<0.001
	≤3.73	458	162	296		458	105	353	
	>3.73	610	151	459		610	45	565	
SII					<0.001				<0.001
	≤918	857	222	635		211	68	143	
	>918	211	91	120		857	82	775	

*ESCC, esophageal squamous cell carcinoma; BMI, body mass index; PNI, prognostic nutritional index; N, number; RT, radiotherapy; T, tumor; N, node; TNM, tumor-node-metastasis; PLR, platelet-to-lymphocyte ratio; NLR, neutrophil-to-lymphocyte ratio; LMR, lymphocyte-to-monocyte ratio; SII, systemic immune-inflammation index.*

### Univariate and Multivariate Survival Analysis of Overall Survival in Esophageal Squamous Cell Carcinoma

**FIGURE 1:**
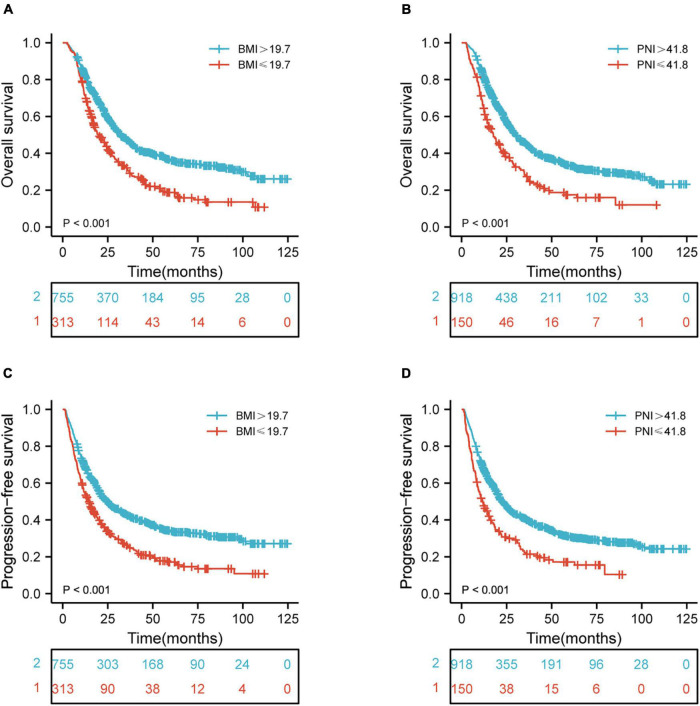
Kaplan-Meier curves of BMI and PNI for the 1,068 patients showing **(A,B)** overall survival (*p* < 0.001, *p* < 0.001, respectively); **(C,D)** progression-free survival (*p* < 0.001, *p* < 0.001, respectively). BMI, body mass index; PNI, prognostic nutritional index.

The median follow-up period for OS was 22.9 months (2.0–124.7 months). Univariate and multivariate Cox regression models for predictors of OS are shown in Table 2. Tumor location (*p* = 0.001), chemotherapy (*p* < 0.001), RT dose (*p* < 0.001), T stage (*p* = 0.049), N stage (*p* < 0.001), TNM stage (*p* < 0.001), BMI (*p* < 0.001), PNI (*p* < 0.001), PLR (*p* < 0.001), NLR (*p* < 0.001), LMR (*p* < 0.001), and SII (*p* < 0.001) were the statistically significant risk variables for a worse OS according to the univariate analysis. The chemotherapy [*p* < 0.001; hazard ratio (HR), 1.447; 95% confidence interval (CI), 1.218–1.719], RT dose (*p* = 0.002; HR, 1.370; 95% CI, 1.128–1.666), N stage (*p* < 0.001; HR, 1.517; 95% CI, 1.272–1.809), TNM stage (*p* = 0.014; HR, 1.360; 95% CI, 1.065–1.738), BMI (*p* < 0.001; HR, 1.355; 95% CI, 1.144–1.605), and PNI (*p* = 0.045; HR, 1.270; 95% CI, 1.005–1.604) were independently associated with a worse OS according to the multivariate analysis. The results indicated that OS was significantly correlated with BMI and PNI in patients with ESCC. The Kaplan-Meier curves of BMI and PNI for OS are shown in Figures 1A,B. Also, the 5-year OS rates were 18.8 and 36.4% for a BMI ≤ 19.7 and a BMI > 19.7, respectively. The 5-year OS rates were 17.5 and 33.4% for a PNI ≤ 41.8 and a PNI > 41.8, respectively.

**TABLE 2:** Predictors of overall survival: univariate and multivariate Cox proportional hazards models.

Clinicopathologic parameters	Univariate analysis			Multivariate analysis		
		
	HR	95% CI	*p*	HR	95% CI	*p*
**Age (years)**						
>70 vs. ≤70	1.122	0.958–1.313	0.152	–		
**Gender**						
Male vs. female	1.106	0.931–1.314	0.252	–		
**Tumor location**						
Cervical/upper vs. middle/lower	0.764	0.650–0.897	0.001	0.893	0.756–1.054	0.179
**Weight loss**						
Yes vs. no	1.025	0.881–1.193	0.749	–		
**Chemotherapy**						
No vs. yes	1.387	1.177–1.634	<0.001	1.447	1.218–1.719	<0.001
**RTdose (Gy)**						
≤59.5 vs. >59.5	1.525	1.257–1.849	<0.001	1.370	1.128–1.666	0.002
**T stage**						
T4 vs. T2/T3	1.165	1.001–1.356	0.049	1.060	0.892–1.259	0.511
**N stage**						
N2/N3 vs. N0/N1	1.760	1.499–2.067	<0.001	1.517	1.272–1.809	<0.001
**TNM stage**						
Stage III/Stage IVA vs. Stage II	1.632	1.328–2.006	<0.001	1.360	1.065–1.738	0.014
**BMI**						
≤19.7 vs. >19.7	1.658	1.414–1.944	<0.001	1.355	1.144–1.605	<0.001
**PNI**						
≤41.8 vs. >41.8	1.733	1.416–2.121	<0.001	1.270	1.005–1.604	0.045
**PLR**						
>195.29 vs. ≤195.29	1.539	1.280–1.851	<0.001	1.053	0.835–1.328	0.661
**NLR**						
>4.56 vs. ≤4.56	1.675	1.336–2.100	<0.001	0.995	0.750–1.319	0.971
**LMR**						
≤3.73 vs. >3.73	1.432	1.230–1.666	<0.001	1.170	0.990–1.381	0.065
**SII**						
>918 vs. ≤918	1.669	1.397–1.994	<0.001	1.268	0.993–1.619	0.057

*HR, hazard ratio; CI, confidence interval; RT, radiotherapy; T, tumor; N, node; TNM, tumor-node-metastasis; BMI, body mass index; PNI, prognostic nutritional index; PLR, platelet-to-lymphocyte ratio; NLR, neutrophil-to-lymphocyte ratio; LMR, lymphocyte-to-monocyte ratio; SII, systemic immune-inflammation index.*

### Univariate and Multivariate Survival Analysis of Progression-Free Survival in Esophageal Squamous Cell Carcinoma

The median follow-up period of PFS was 17.4 months (1.1–124.7 months). Table 3 shows the univariate and multivariate Cox regression models for predictors of PFS. Tumor location (p = 0.005), chemotherapy (*p* = 0.001), RT dose (*p* = 0.001), T stage (*p* = 0.015), N stage (*p* < 0.001), TNM stage (*p* < 0.001), BMI (*p* < 0.001), PNI (*p* < 0.001), PLR (*p* < 0.001), NLR (*p* < 0.001), LMR (*p* < 0.001), and SII (*p* < 0.001) were shown to be significantly associated with a worse PFS according to the univariate analysis. In multivariate analysis, the chemotherapy (*p* < 0.001; HR, 1.378; 95% CI, 1.163–1.633), RT dose (*p* = 0.018; HR, 1.261; 95% CI, 1.040–1.529), N stage (*p* < 0.001; HR, 1.522; 95% CI, 1.281–1.808), TNM stage (*p* = 0.012; HR, 1.362; 95% CI, 1.071–1.731), BMI (*p* < 0.001; HR, 1.354; 95% CI, 1.148–1.597), PNI (*p* = 0.019; HR, 1.308; 95% CI, 1.044–1.638), and SII (*p* = 0.044; HR, 1.277; 95% CI, 1.007–1.618) were independently associated with a worse PFS. These results indicated that PFS was significantly correlated with BMI and PNI in ESCC patients. The Kaplan-Meier curves of BMI and PNI for PFS are shown in Figures 1C,D. Also, the 5-year PFS rates were 17.2 and 34.0% for a BMI ≤ 19.7 and a BMI > 19.7, respectively. The 5-year PFS rates were 17.2 and 30.9% for a PNI ≤ 41.8 and a PNI > 41.8, respectively.

**TABLE 3:** Predictors of progression-free survival: univariate and multivariate Cox proportional hazards models.

Clinicopathologic parameters	Univariate analysis			Multivariate analysis		
		
	HR	95% CI	*p*	HR	95% CI	p
**Age (years)**						
>70 vs. ≤70	1.068	0.915–1.246	0.405	–		
**Gender**						
Male vs. female	1.061	0.975–1.154	0.172	–		
**Tumor location**						
Cervical/upper vs. middle/lower	0.798	0.683–0.933	0.005	0.912	0.776–1.071	0.259
**Weight loss**						
Yes vs. no	1.059	0.913–1.228	0.448	–		
**Chemotherapy**						
No vs. yes	1.302	1.109–1.530	0.001	1.378	1.163–1.633	<0.001
**RTdose (Gy)**						
≤59.5 vs. >59.5	1.394	1.152–1.687	0.001	1.261	1.040–1.529	0.018
**T stage**						
T4 vs. T2/T3	1.202	1.036–1.394	0.015	1.087	0.918–1.287	0.333
**N stage**						
N2/N3 vs. N0/N1	1.738	1.485–2.033	<0.001	1.522	1.281–1.808	<0.001
**TNM stage**						
Stage III/stage IVA vs. stage II	1.673	1.367–2.047	<0.001	1.362	1.071–1.731	0.012
**BMI**						
≤19.7 vs. >19.7	1.639	1.402–1.915	<0.001	1.354	1.148–1.597	<0.001
**PNI**						
≤41.8 vs. >41.8	1.712	1.404–2.087	<0.001	1.308	1.044–1.638	0.019
**PLR**						
>195.29 vs. ≤195.29	1.540	1.285–1.845	<0.001	1.067	0.853–1.335	0.571
**NLR**						
>4.56 vs. ≤4.56	1.691	1.354–2.112	<0.001	1.042	0.794–1.368	0.768
**LMR**						
≤3.73 vs. >3.73	1.413	1.218–1.640	<0.001	1.146	0.974–1.349	0.100
**SII**						
>918 vs. ≤918	1.688	1.418–2.009	<0.001	1.277	1.007–1.618	0.044

*HR, hazard ratio; CI, confidence interval; RT, radiotherapy; T, tumor; N, node; TNM, tumor-node-metastasis; BMI, body mass index; PNI, prognostic nutritional index; PLR, platelet-to-lymphocyte ratio; NLR, neutrophil-to-lymphocyte ratio; LMR, lymphocyte-to-monocyte ratio; SII, systemic immune-inflammation index.*

### A Prognostic Nomogram Based on Pre-therapeutic Nutritional Indices

**FIGURE 2:**
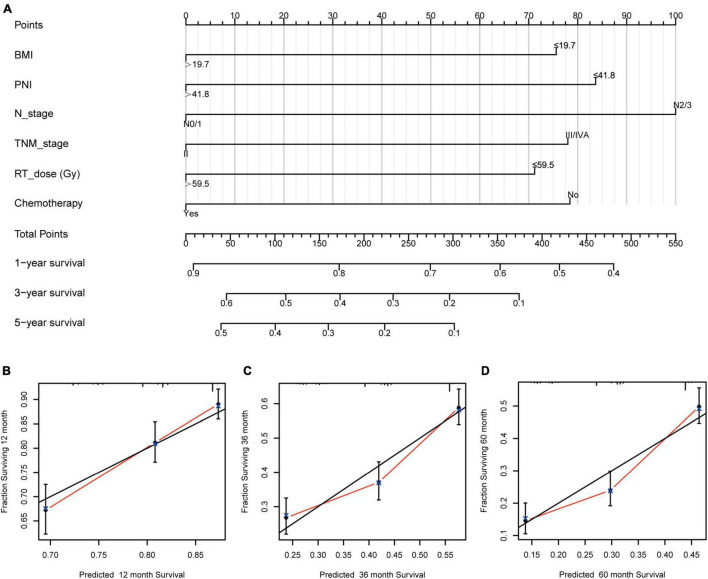
Nomogram and calibration curve for predicting the probability of OS for the 1,068 patients showing **(A)** a nomogram that integrates BMI, PNI, N stage, TNM stage, RT dose, and chemotherapy in ESCC patients; **(B–D)** the calibration curve of the nomogram. OS, overall survival; BMI, body mass index; PNI, prognostic nutritional index; N, node; TNM, tumor-node-metastasis; RT, radiotherapy; ESCC, esophageal squamous cell carcinoma.

The independent prognostic factors for ESCC were chemotherapy, RT dose, N stage, TNM stage, BMI, and PNI. A prognostic nomogram for OS (Figure 2) was constructed. A worse prognostic factor is indicated by a higher score. With 1,000 bootstrap resamples for the nomogram, the C-index was 0.63.

### Risk Stratification of Nutritional Indices

**FIGURE 3:**
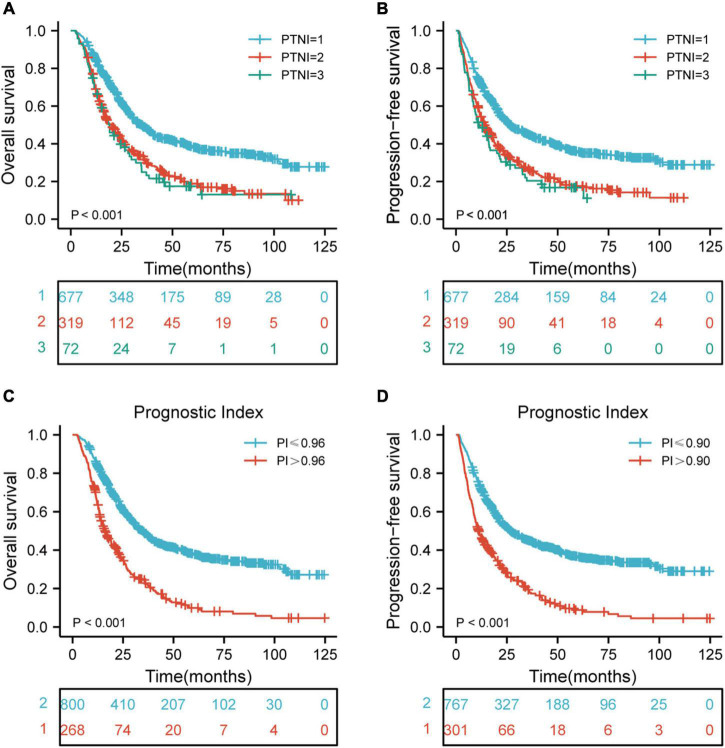
Risk stratification for PTNI and PI on OS and PFS according to risk groups. **(A)** Risk stratification for PTNI on OS (*p* < 0.001); **(B)** risk stratification for PTNI on PFS (*p* < 0.001); **(C)** risk stratification for PI on OS (*p* < 0.001); **(D)** risk stratification for PI on PFS (*p* < 0.001); OS, overall survival; PFS, progression-free survival; PTNI, pre-therapeutic nutritional index; PI, prognostic index.

BMI and PNI were found to be significant prognostic factors for both OS and PFS in univariate Cox regression analysis. Furthermore, BMI and PNI were found to be independent prognostic factors affecting both OS and PFS. As a result of these two risk factors, the patients were further categorized into three groups using pre-therapeutic BMI and PNI values: low-risk group: pre-therapeutic nutritional index (PTNI) score = 1 (neither a low BMI nor a low PNI); medium-risk group: PTNI score = 2 (either a low BMI or a low PNI); high-risk group: PTNI score = 3 (both a low BMI and a low PNI), with 5-year OS rates of 38.5, 18.9, 17.5%, respectively (*p* < 0.001, Figure 3A) and 5- year PFS rates of 35.8, 17.6, 16.8%, respectively (*p* <0.001, Figure 3B). Besides, we also constructed a risk prediction model for OS and PFS. The prognostic index (PI) was calculated based on statistically significant factors for predicting OS and PFS. Each risk factor (one for existence or zero for absence) was multiplied by a b coefficient according to the result of multivariate survival analysis. The PI of OS was calculated as 0.315*RT dose + 0.369*chemotherapy + 0.417*N stage + 0.308*TNM stage + 0.239*PNI + 0.304*BMI. The PI of PFS was calculated as 0.232* RT dose + 0.321*chemotherapy + 0.42*N stage + 0.309*TNM stage + 0.269*PNI + 0.303*BMI + 0.244*SII. The calculated PI was dichotomized by 0.96 to obtain 268 high-risk and 800 low-risk subsets of OS. Similarly, the calculated PI was dichotomized by 0.90 to obtain 301 high-risk and 767 low-risk subsets of PFS. Finally, the results revealed that the high-risk group had worse OS and PFS than the low-risk group (*p* < 0.001, Figures 3C,D).

### Kaplan-Meier Curves of Various Risk Groups Based on Clinical Stage

**FIGURE 4:**
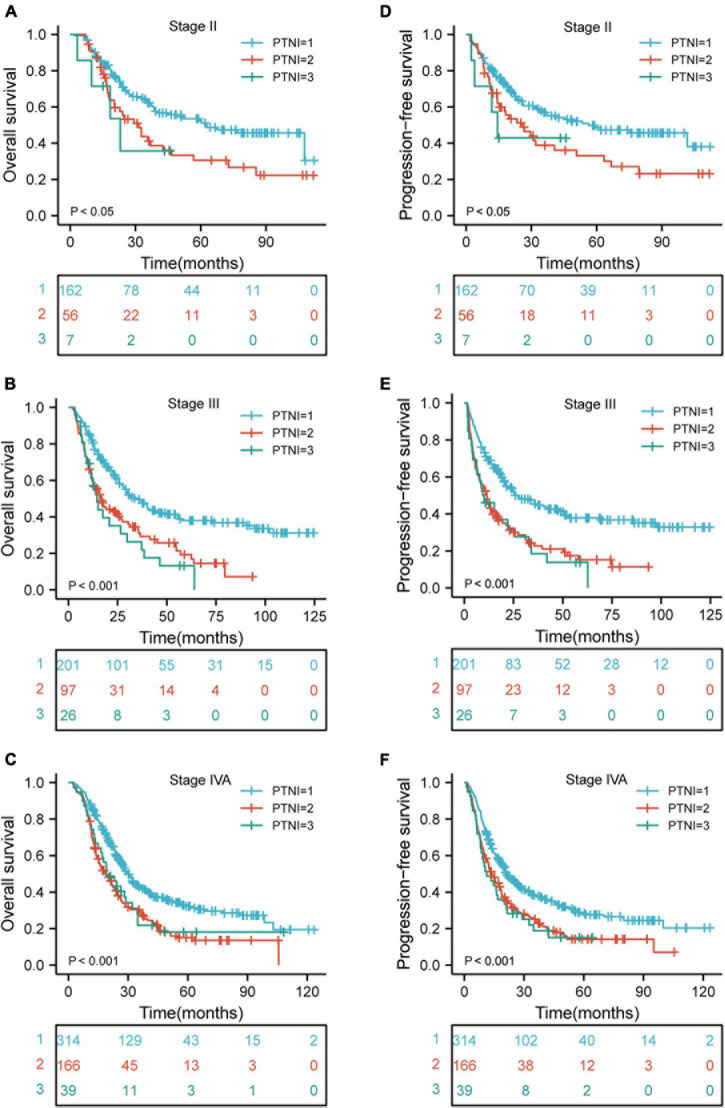
Kaplan-Meier curves according to TNM stage categories for the 1,068 patients showing **(A–F)** overall survival (*p* < 0.05, *p* < 0.001, *p* < 0.001, respectively) of patients with TNM stage II, III, and IVA; progression-free survival (*p* < 0.05, *p* < 0.001, *p* < 0.001, respectively) of patients with TNM stage II, III, and IVA. TNM, tumor-node-metastasis; PTNI, pre-therapeutic nutritional index.

There were significant differences in OS rates for ESCC patients at clinical stages II, III, and IVA (*p* < 0.05, *p* < 0.001, and *p* < 0.001, respectively) when comparing the rates in different risk groups. Also, a comparison of the PFS rates in different risk groups revealed significant differences for patients with ESCC who are at clinical stages II, III, and IVA (*p* < 0.05, *p* < 0.001, and *p* < 0.001, respectively) (Figures 4A–F).

### Survival Stratified by Different Nutritional Risks With or Without Chemotherapy

**FIGURE 5:**
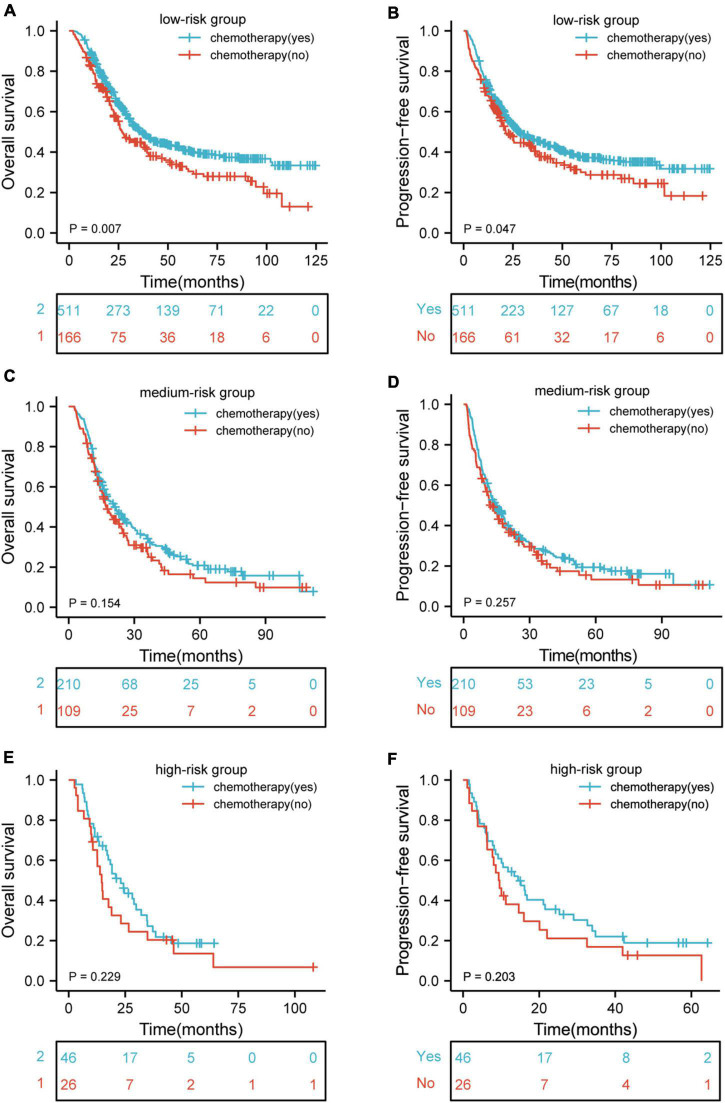
Kaplan–Meier curves according to patients who receive and did not receive chemotherapy showing **(A,B)** overall survival (*p* = 0.007) and progression-free survival (*p* = 0.047) for patients receive and did not receive chemotherapy in low-risk group; **(C,D)** overall survival (*p* = 0.154) and progression-free survival (*p* = 0.257) for patients receive and did not receive chemotherapy in medium-risk group; **(E,F)** overall survival (*p* = 0.229) and progression-free survival (*p* = 0.203) for patients receive and did not receive chemotherapy in high-risk group. HR, hazard ratio.

In order to verify whether the poor long-term prognosis of patients with high nutritional risk is due to poor tolerance to chemotherapy and low completion rate of treatment. We further analyzed the effects of chemotherapy on OS and PFS in different nutritional risk groups. As shown in Figures 5A,B, patients who received chemotherapy had better OS (*p* = 0.007) and PFS (*p* = 0.047) than those who did not receive chemotherapy in low-risk group. In addition, Figures 5C–F showed that there were no significant differences in OS and PFS among the patients who receive or did not receive chemotherapy in medium-risk and high-risk groups (*p* > 0.05 for all).

### The Relationship Between the Nutritional Indices and Survival

**FIGURE 6:**
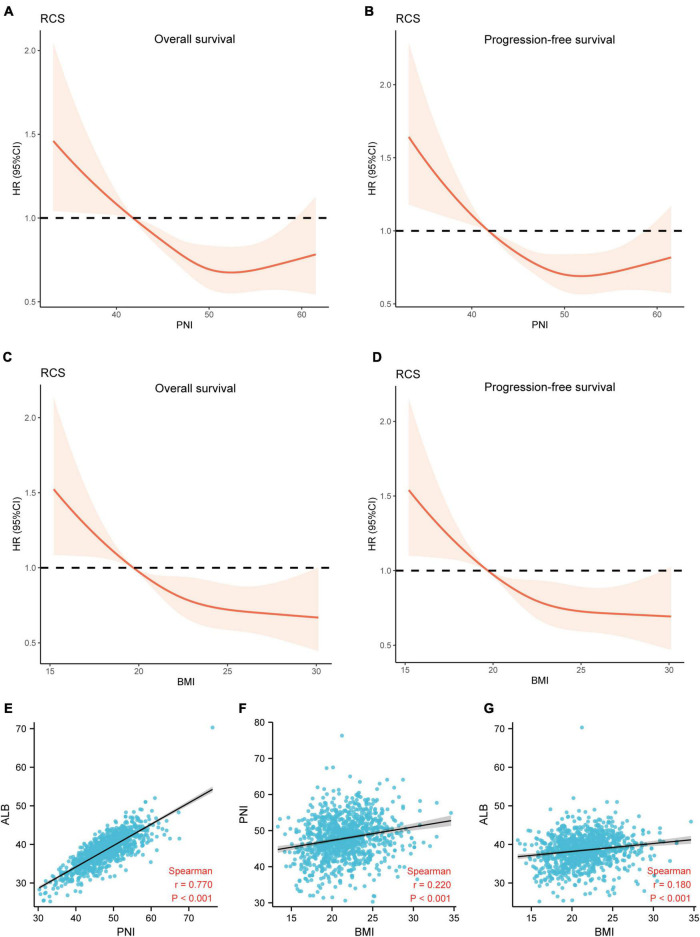
The relationship between the nutritional indices and survival. **(A,B)** A non-linear relationship between the PNI and survival for patients with ESCC. The death hazard of PNI sharply decreased at 41.8; **(C,D)** a linear relationship between the BMI and survival, with an inferior OS and PFS when a decrease in the PLR at 19.7; **(E)** the correlation between PNI and ALB (Cor = 0.770, *p* < 0.001); **(F)** the correlation between BMI and PNI (Cor = 0.220, *p* < 0.001); **(G)** the correlation between BMI and ALB (Cor = 0.180, *p* < 0.001). OS, overall survival; PFS, progression-free survival; PNI, prognostic nutritional index; ALB, albumin; Cor, correlation; BMI, body mass index.

Restricted cubic spline (RCS) analysis was used to classify the association between nutritional indices and survival. Figures 6A–D demonstrated a non-linear relationship between the PNI and OS as well as PFS for patients with ESCC. The death hazard of PNI sharply decreased to 41.8. Nevertheless, for BMI, it presented a linear relationship between the BMI and survival, with an inferior OS and PFS when a decrease in the BMI. Spearman’s correlation was used to analyze the correlation between the nutritional indices. The results indicated that PNI is positively correlated with ALB (Cor = 0.770, *p* < 0.001) and BMI (Cor = 0.220, *p* < 0.001). Also, BMI also demonstrated to be positively correlated with ALB (Cor = 0.180, *p* < 0.001) (Figures 6E–G).

## Discussion

EC is still a major challenge with poor survival rates due to its high malignant potential. Patients with digestive tract cancers were more likely to be affected by malnutrition (21). It was reported that more than 60% of patients with EC were malnourished (22). Radiotherapy plays a vital role in the multimodal treatment of EC. However, the incidence of malnutrition in patients with EC who are treated with RT is estimated to be more than 50% (23). In addition, malnutrition in patients with EC may worsen during RT, leading to some patients discontinuing treatment. Worse, several studies have suggested that malnutrition may increase mortality and morbidity after chemoradiotherapy (24, 25). Pre-therapeutic identification of high-risk patients using a simple index is therefore clinically important. The TNM stage was considered the main predictor of prognosis. Nevertheless, there is still prognostic heterogeneity among patients with the same TNM stage (26). An accurate prognostic index, in addition to the current TNM stage system, will be extremely useful in guiding individual treatment strategies.

Mounting evidence has demonstrated that inflammatory and nutritional indices are significantly associated with clinicopathological parameters and prognosis in EC patients (3, 4, 9, 10). Given the above, retrospective analysis to determine associations between pre-therapeutic inflammatory and nutritional indices and survival outcomes in patients with ESCC who received dCRT was performed. Immunological status including inflammatory status and nutritional status. Many markers of nutritional status are assessed by lymphocyte count. This indicates that the inflammatory status is closely related to the nutritional status. Taken together, we supposed that the inflammatory and nutritional status of cancer patients may affect the prognosis through immunity. Therefore, in our study, we used the available data to include the inflammatory indices (PLR, NLR, LMR, and SII) and the nutritional indices (BMI and PNI) together in the analysis. Inflammatory indices were the statistically significant risk variables for a worse OS and PFS according to the univariate analysis. Nonetheless, these indices were not statistically significant in multivariate analysis. In addition, the results revealed that both BMI and PNI are independent prognostic factors for OS and PFS in ESCC. A nomogram model to predict the prognosis of patients was also constructed. This will help clinicians assess the patient’s prognosis after dCRT. No previous study has evaluated the risk stratification of BMI and PNI in patients with ESCC who received dCRT.

There is still no consensus about the most useful and best nutritional index for the prognostic evaluation of patients with locally advanced ESCC who are treated with dCRT. Previous studies have demonstrated BMI to be useful for predicting survival outcomes of patients with some cancers (11–13), including ESCC (3, 4). However, EC is a type of tumor entity in which being overweight is rare (4, 27). Therefore, the assessment based on BMI seems reasonable (6). The BMI was selected as the nutritional index because it can be easily obtained. A BMI cutoff value of 19.7 kg/m^2^ was used for the identification of ESCC patients with pre-therapeutic risk. This cutoff value of BMI is different from other studies. The heterogeneity between the studies and the different sample sizes are the main reasons for the inconsistency of the research results.

PNI is another prognostic index that has been widely evaluated in recent years. The serum ALB level (g/L) + 5 multiplied by the absolute lymphocyte count to calculate the PNI. ALB is associated with cancer prognosis. The optimal cutoff value for PNI was calculated to be 41.8. A decrease in the count of lymphocytes and an impairment in lymphocyte function will lead to a worse prognosis in cancer patients (28). Therefore, PNI could quantify the inflammatory and nutritional status of patients with ESCC. Since PNI is easily obtained before treatment (29), it can reflect the inflammatory and nutritional status of cancer patients, help predict the prognosis, and guide the treatment of patients with ESCC. Previous studies have also shown that PNI is an essential prognostic index in patients with cervical ESCC (30). However, due to the differences in research samples and various PNI categories, obtaining a consistent definition across studies is difficult. The association between PNI and survival outcomes remains controversial.

The prognosis of cancer patients depended not only on the tumor but also on the host factors. As is known to all, the tumor stage significantly affected the prognosis of EC patients. In addition, inflammatory and nutritional status also played important role in patients with EC (3, 4, 9, 10). Nonetheless, all the inflammation and nutrition assessment methods had some limitations. In the absence of a consensus, the combination of inflammatory and nutritional indices and other risk factors is often considered a reliable method of assessing survival outcomes. This study used inflammatory and nutritional indices and clinicopathological factors including BMI, PNI, chemotherapy, RT dose, N stage, and TNM stage to construct a nomogram. The result suggests that this combination is beneficial for selecting the best clinical therapy and ultimately improving the survival outcomes of patients.

The mechanism by which nutritional status affects survival outcomes has not been fully described. The potential explanations were: First, malnutrition inhibits the development and maturation of the immune system (31). Second, malnutrition may impact patients’ tolerance to active treatments (32). Third, malnutrition impairs the activation of nutrients in the immune system and alters the immune regulation of the intestinal flora (33). To better understand the prognostic role of nutritional indices, a novel prognostic index named PTNI was developed. It was determined using the cutoff value and could categorize patients into three risk groups. The corresponding 5-year OS rates were 38.5, 18.9, and 17.5% (*p* < 0.001) and 5-year PFS rates were 35.8, 17.6, and 16.8% (*p* < 0.001) in the low-risk, medium-risk, and high-risk groups, respectively. Furthermore, the survival outcomes of various risk groups were analyzed according to the clinical stage. The results revealed that there were significant differences in OS and PFS for ESCC patients with clinical stages II, III, and IVA in the various risk groups.

RT is a momentous treatment option for locally advanced ESCC. However, radiation esophagitis (RE) is one of the most common adverse reactions in RT for ESCC. When RE occurs, it aggravates malnutrition and thus reduces the effectiveness of therapy (34, 35). The study implies that increased nutritional risk is related to reduced survival outcomes in patients with locally advanced ESCC who are treated with dCRT. The study also suggests that interventions are necessary for patients with ESCC who have low BMI and low PNI. The pre-therapeutic evaluation of malnutrition may improve the prognosis in ESCC patients who receive dCRT (36). Lifestyle modification, including exercise and dietary changes, improves the nutritional status of cancer patients (37). Furthermore, nutrition intervention could reduce the side effects of antitumor drugs (35, 38–40). However, the effect of pre-therapeutic interventions on ESCC patients with low BMI and low PNI needs to be further evaluated.

An increasing body of evidence showed that PET can provide independent prognostic information for patients with EC (41, 42). PET is a functional imaging modality that can predict EC prognosis more accurately than conventional CT (43). In most cases, SUVmax (maximum standardized uptake value), MTV (metabolic tumor volume), and TLG (total lesion glycolysis) are commonly used parameters in PET to predict the prognosis of tumor patients. This has been proven in some studies (44, 45). The biological status of tumors is constantly changing, and PET can detect tumor metabolic responses earlier than CT. Therefore, PET seems to be a particularly useful tool for predicting outcomes for EC patients. Several previous studies have shown that continuous PET monitoring during treatment can predict the outcome of chemotherapy and radiotherapy in EC patients (46). Nonetheless, due to the high cost of PET examination, there are some patients who may only have a PET examination once or not at all. That makes the PET examination a bit limited. Therefore, researchers need to continue to explore the prognostic value of PET parameters. More useful PET parameters are needed to predict the prognosis of EC patients right now. In addition, some studies have also indicated that other indices at the time of diagnosis might be associated with prognosis. Geng et al. indicated that the systemic inflammation response index (SIRI), as an inflammatory index of EC, is a new prognostic index and therapeutic response monitor for EC patients (47). The serum albumin and C-reactive protein (CRP) are biomarkers representing nutritional status and systemic inflammation, respectively. Low albuminemia and high CRP level are associated with poor outcomes in EC patients (48). Some studies have demonstrated the prognostic value of C-reactive protein-albumin ratio (CAR) in EC patients. Wang et al. revealed that EC patients with lower pretreatment CAR had better OS (49). However, further large-scale studies with bigger samples are still needed to validate these findings.

There are some limitations to the study that should be mentioned. Firstly, it was a retrospective study at a single institution. Biases in the data collection process could have an impact on the study conclusions. Secondly, due to a lack of relevant data, other inflammation and nutritional indices were not studied. Besides, these indices might be associated with each other. The T stage and N stage may be also related to TNM stage. Whether these variables can be incorporated into covariates for multivariate analysis remains to be clarified. Thirdly, there could have been a selection bias since the study included patients who did not receive chemotherapy as well as those who did. Our study is limited to patients with locally advanced ESCC undergoing dCRT and has no guiding significance for patients with locally advanced ESCC undergoing neoadjuvant therapy or adjuvant therapy. Fourthly, the starting time point for both OS and PFS was pathologic confirmation of the disease. The attendance date to radiotherapy department was set to collect BMI and for biochemical data needed to calculate PNI. Whether this time gap could affect nutrition status of the participants still needs further verification. Finally, other institutions should verify the proposed BMI and PNI cutoff values due to other confounding factors that may influence the values of BMI and PNI. Larger prospective studies with standard assessing and well-designed methods are required in the future to confirm the current findings due to the aforementioned limitations.

## Conclusion

In conclusion, comparative analyses for associations between pre-therapeutic inflammation and nutritional indices and survival outcomes were carried out in patients with locally advanced ESCC who received dCRT. The decreased pre-therapeutic BMI and PNI levels were associated with a worse prognosis and independently predicted OS and PFS. BMI and PNI are easy to obtain and can be used to stratify risk factors for ESCC patients. Further prospective studies with large cohorts are needed to offer optimal treatment strategies for patients with locally advanced ESCC receiving dCRT and obtain more definitive results.

## Data Availability Statement

The raw data supporting the conclusions of this article will be made available by the authors, without undue reservation.

## Ethics Statement

The current study was approved by the Ethics Committee of Fujian Medical University Cancer Hospital, Fuzhou, China, and conducted in accordance with the principles of the Declaration of Helsinki and its amendment. All patients provided written informed consent prior to treatment, and all the information was anonymized prior to analysis.

## Author Contributions

WC, QY, JL, XC, YY, and HW designed the study. YY, JQ, DK, YW, ML, TL, QZ, HL, and ZW contributed to the data collection. XC, YY, JQ, DK, YW, and ML analyzed the data. WC, JL, QY, XC, and HW supervised the study. XC, YY, HZ, JQ, DK, ML, JY, and LL wrote the manuscript. All authors reviewed and approved the final manuscript.

## Conflict of Interest

The authors declare that the research was conducted in the absence of any commercial or financial relationships that could be construed as a potential conflict of interest.

## Publisher’s Note

All claims expressed in this article are solely those of the authors and do not necessarily represent those of their affiliated organizations, or those of the publisher, the editors and the reviewers. Any product that may be evaluated in this article, or claim that may be made by its manufacturer, is not guaranteed or endorsed by the publisher.

## Footnotes

1
https://medicine.yale.edu/lab/rimm/research/software/

## Abbreviations

ESCC: esophageal squamous cell carcinoma
dCRT: definitive chemoradiotherapy
OS: overall survival
PFS: progression-free survival
EC: esophageal carcinoma
BMI: body mass index
PNI: prognostic nutrition index
KPS: karnofsky performance status
T: tumor
N: node
TNM: tumor-node-metastasis
CT: computed tomography
3D-CRT: three dimensional-conformal radiotherapy
IMRT: intensity modulated radiotherapy
PTV: planned target volume
ALB: albumin
PLR: platelet-to-lymphocyte ratio
NLR: neutrophil-to-lymphocyte ratio
PLR: platelet-to-lymphocyte ratio
SII: systemic immune-inflammation index
PET-CT: positron emission tomography-CT
HR: hazard ratio
CI: confidence interval
PTNI: pre-therapeutic nutritional index
Cor: correlation
ROC: receiver-operating characteristic
RCS: restricted cubic splines
PI: prognostic index
SUVmax: maximum standardized uptake value
MTV: metabolic tumor volume
TLG: total lesion glycolysis
SIRI: systemic inflammation response index
CRP: C-reactive protein
CAR: C-reactive protein-albumin ratio.
